# Utopia for Norwegian helicopter emergency medical services: Estimating the number of bases needed to radically bring down response times, and lives needed to be saved for cost effectiveness

**DOI:** 10.1371/journal.pone.0281706

**Published:** 2023-03-30

**Authors:** Caroline Jeanne Jagtenberg, Oddvar Uleberg, Gudrun Maria Waaler Bjørnelv, Jo Røislien

**Affiliations:** 1 Department of Operations Analytics, School of Business and Economics, Vrije Universiteit Amsterdam, Amsterdam, The Netherlands; 2 Department of Emergency Medicine and Pre-hospital Services, St. Olav`s University Hospital, Trondheim, Norway; 3 Division of Emergencies and Critical Care, Department of Research and Development, Oslo University Hospital, Oslo, Norway; 4 Department of Public Health and Nursing, Norwegian University of Science and Technology, Trondheim, Norway; 5 Department of Health Management and Health Economics, Institute of Health and Society, Faculty of Medicine, University of Oslo, Oslo, Norway; 6 Department of Research, Norwegian Air Ambulance Foundation, Oslo, Norway; 7 Faculty of Health Sciences, University of Stavanger, Stavanger, Norway; Aalborg University and Aalborg University Hospital, DENMARK

## Abstract

**Objectives:**

Helicopter Emergency Medical Services (HEMS) throughout Europe are generally on scene within 10–15 minutes. In Norway, however, with its 13 HEMS bases, only 75% of the population can currently be reached within half an hour. We estimate the number of HEMS bases needed to reach the full Norwegian population within 10–15 minutes, and discuss implications regarding cost effectiveness.

**Methods:**

Using geographic location and population characteristics from Norway’s 428 municipalities as input to the Maximal Covering Location Problem–a mathematical location optimization model–we estimate the number of HEMS bases required along with accompanying personnel and healthcare costs. We estimate the minimum number of lives that would have to be saved to achieve a net social benefit of zero.

**Results:**

To reach 99% or 100% of the Norwegian population by HEMS within 15 minutes 78 or 104 bases are needed, respectively. The incremental need for personnel going from 20 to 15 minutes for 99/100% of the population is 602/728, with an accompanying incremental cost of 228/276 million EURO per year. A yearly total of 280/339 additional lives would have to be saved to obtain a net social benefit of zero. Then, the HEMS-system as a whole would be cost effective although the least efficient bases still would not be.

**Conclusions:**

Reducing Norwegian HEMS response times to 10–15 minutes requires a drastic increase in the number of HEMS bases needed. Choice of ethical philosophy (utilitarianism or egalitarianism) determines when the expansion might be considered cost effective.

## Introduction

Helicopter Emergency Medical Services (HEMS) have expanded worldwide, and is now a fundamental part of many healthcare systems. While HEMS is a costly service, and its efficiency is continuously debated [1], the service provides several advantages such as advanced point-of-care diagnostics, multifaceted clinical decision making, access to remote locations and advanced interventions beyond the scope of most ground-based Emergency Medical Sercvices (EMS) [2, 3]. The service is resource intensive, and there is a constant push to optimize the number and location of HEMS bases with respect to external targets, such as response times and available funds [4–6].

The response time–the time from the emergency call to the HEMS on-scene arrival–is often used as a measure of the service’s performance. Faster response times are often seen as indicative of better care, and several EMS and HEMS providers have converted response times into specific targets [7]. Such targets can often be traced back to the work done by Eisenberg et al. in 1979, who observed better outcomes in patients with out-of-hospital-cardiac-arrest (OHCA) when cardiopulmonary resuscitation and defibrillation were initiated rapidly [8]. However, OHCA accounts for just 1–2% of actual EMS missions and there is still scarce knowledge of the actual effects of reduced response times in conditions other than urban trauma and cardiac arrest [7, 9]. Regardless, substantial investments have been made to reduce response times in EMS.

In several European countries (e.g Germany, Austria and Switzerland) physician-manned EMS/HEMS operate with response-time targets between 10 and 15 minutes [10–12]. In Norway, however, with its large sparsely-populated areas and long travel distances, response times are significantly longer. HEMS is considered essential to provide equal access to specialized healthcare throughout the country, regardless of residential pattern, with a so-called “no man left behind” strategy [13]. In Norway, equal access to care is a pivotal principle, and a 2001 government white paper stated that 90% of the population should be reached by a physician-manned EMS/HEMS within 45 minutes [14]. An integrated HEMS is considered as having a compensatory effect that adjusts for the potential unequal access to advanced emergency medical care due to the geographic spread [13].

Norway currently has 13 HEMS bases, established through historical local engagement from the late 1970s, with gradual expansion covering an increasing part of Norway [15]. Several studies have used mathematical models to study the optimal location of HEMS bases in Norway based on minor adjustments to the existing resources [3, 5, 15]. However, what it takes to bring response times in Norway down to times comparable to those on mainland Europe remains unexamined.

From a global perspective, there is scarce research combining cost-effectiveness analysis with HEMS expansion. Most cost-effectiveness studies are done retrospectively, after the bases are already operational [16]. The few exceptions that do attempt to estimate cost effectiveness of unimplemented changes either focus on a specific disease [17, 18] or scenarios that differentiate between HEMS operating hours [19]–but not base locations. To the best of our knowledge, no one has studied whether a potentially drastic increase in the number of HEMS bases would be cost effective.

While reducing response times would be beneficial from a medical viewpoint, it will also bring increased financial and safety costs [7]. When establishing priorities in Norway, resource demand and cost should be considered together with the expected effect of a treatment and the severity of the condition (typically in cost-effectiveness analyses) [20].

The primary aim of the present study was to estimate the number of HEMS bases needed in order to reach the Norwegian population within response-time targets that match those of countries in continental Europe. We used a mathematical model, systematically varying the proportion of the population reached and the response times required. Our secondary aim was to estimate the need for personnel and expected healthcare costs given the expansions, and consequently, to estimate the necessary effect required (i.e., the number of lives needed to save) to achieve a net social benefit of zero, given nationally acceptable values of a statistical life. The intention of this work is to bring the field a step closer to more informed decision making regarding HEMS expansion.

## Methods

### Study setting

**Fig 1:**
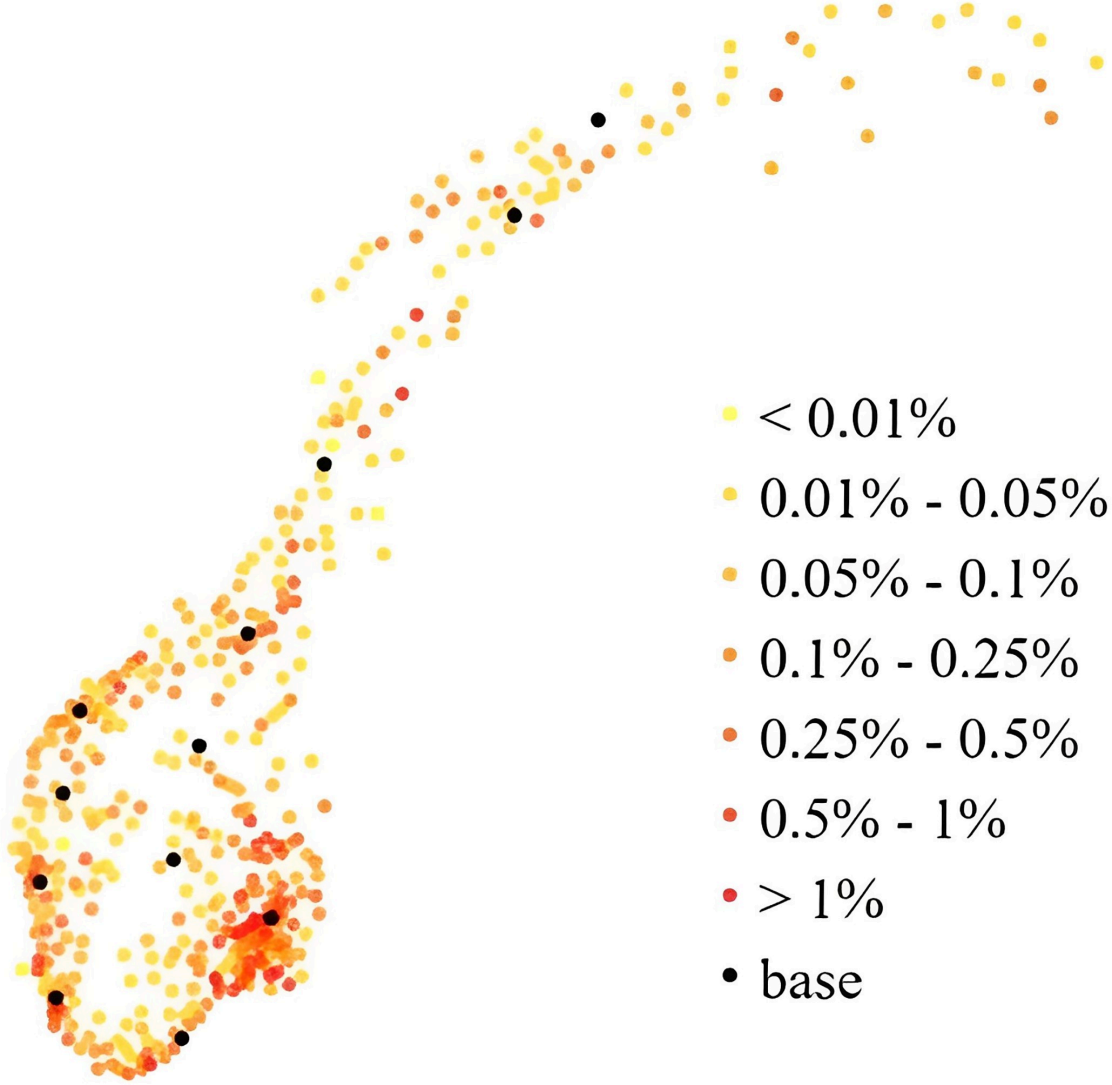
The 428 municipalities in Norway in 2015. The color indicates the proportion of the population that lives there, and the 12 bases that existed in 2015.

The Norwegian mainland covers 323,780 km^2^ at the far North of Europe, stretching 1790 km from north to south. In 2015, the population was 5.2 million [21], with county population density ranging from only 1.5 inhabitants/km^2^ in the northernmost county Finnmark to 1129.5 inhabitants/km^2^ in Oslo. For mathematical modelling of HEMS base locations in Norway, the difference between using municipality data and fine grid data is negligible [15] and the present study uses municipality level population data as this drastically reduces computation times. Population data are freely available from Statistics Norway [22]. Following a previous publication [5] we used the 428 municipalities that constituted Norway in 2015, each represented as a population-weighted centroid, see Fig 1.

Norway has a publicly funded National Air Ambulance Service consisting of 12 HEMS bases in 2015 (13 bases from 1^st^ of February 2021). At any given time, a base crew consists of a pilot, a paramedic and a consultant anaesthesiologist. In order to comply with labor laws and regulations, a minimum of 4 pilots, 4 paramedics and 6 anaesthesiologists are stationed at each base to secure 24/7/365 operative capability.

### Mathematical modeling

To estimate the minimal number of HEMS bases needed to meet a certain demand, we applied the Maximal Covering Location Problem (MCLP) [22]; a mathematical optimization model. This model uses as input a budget to build a fixed number of bases throughout a geographical area, and outputs the fraction of patients that can maximally be covered if those bases were in optimal locations. In the MCLP model, a patient is said to be covered if they can be reached from the nearest base within a certain time threshold. This performance would be attained in ideal circumstances: the service is not busy when requested, and the helicopter can fly in a straight line.

To determine how many bases are needed in order to guarantee a certain coverage, we solve the MCLP model multiple times, each time increasing the available budget, until the desired coverage is reached. This approach works well for almost all desired percentages of coverage, except for the extreme case of 100% where a small adjustment is advisable: instead of counting the fraction of covered *population*, count the fraction of covered *municipalities*. If the target is 100%, this adjusted model returns the same base locations as the original model, but is faster to solve.

### Computations

Each of the 428 municipalities in Norway are used as demand locations, where the demand in each municipality is modeled as the fraction of the Norwegian population living there. All 428 municipalities are also used as potential HEMS base locations. We performed both a greenfield analysis, computing optimal base locations as if no current bases exist, and a brownfield analysis, exploring what the optimal addition of bases would be while keeping the 12 bases (existing status in 2015) in Norway. For the brownfield analysis, we report the number of *additional* bases that would have to be built, not including the existing 12. We computed results for a range of different response time targets from 10 to 60 minutes.

We estimated response times from any potential base to any municipality using a 5.5 minute reaction time [13] and an average helicopter speed of 220 km/h [3]. The models were implemented in Python using the PuLP package [23] and solved with CBC [24].

### Estimated medical demand

In 2013, Krüger et al. calculated a population incidence of medical conditions, and the proportion of patients with severely deranged vital signs and/or requiring advanced therapy [25]. They found incidence rates for critical illness and injury of 25–30 per 10,000 person-years [25]. For a current Norwegian population of 5,415,166 [26] that would indicate an estimated need of approximately 16,246 missions per year.

### Costs

The estimated healthcare personnel needed to operate the HEMS bases is computed according to current legislation where a total of 14 crew members would be needed, per base, in order to run the base 24/7/365. This amounts to 4 pilots, 4 paramedics and 6 doctors.

To estimate the healthcare costs, we used information on resource use and costs from the accounting system of current HEMS bases in Norway. The annual healthcare cost of operating one Norwegian helicopter base was estimated as approximately 54 million 2021 Norwegian kroner (NOK) (€5.3 million [27]). This includes the cost of 39.9 million NOK (€3.9 million) for all operative running costs (e.g. fuel, maintenance, salary for pilot and paramedic, and base facilities) [Norwegian Air Ambulance Services, personal communication]. The annual costs of six anesthesiologists and medical equipment was estimated at 14 million 2021 NOK (€1.4 million) [St. Olav`s University Hospital, personal communication].

### Benefits

The net social benefit (NSB) (i.e., the cost-benefit) of the HEMS-expansion can be estimated by subtracting the expected costs from the corresponding expected benefits:

$$NSB_{i}=b_{i}-c_{i}.$$ (1)

Here NSB_i_ is the net social benefit of project i, b_i_ is the benefits and c_i_ the costs. If NSB is positive, the intervention (expansion) would be considered cost beneficial / cost effective.

Unfortunately, the estimated expected benefit of increasing the number of HEMS is unknown, and consequently the expected cost-benefit of increasing the number of HEMS in Norway cannot be estimated according to (equation 1).

However, the expected healthcare costs of the expansion can be estimated using the previously specified numbers. Also, the value placed on saving a life is quantified in Norway and termed *the value of a statistical life* (VSL). By combining this, we can estimate the *necessary* lives needed to save for the expansion to achieve a positive NSB:

Livesneededtosave=Healthcarecosts/VSL (2)

### The value of a statistical life

The VSL is estimated by asking people representing the general population how much they value a reduction in the risk of dying. In our calculations the VSL was set to 8.13 million 2021 NOK (€813,911 [27]), using estimates from the Norwegian Agency for Public and Financial Management and the Norwegian Directorate of Health [28, 29]. Our estimate was based on a value of 30 million NOK 2012 (€3.0 million [27]) for a VSL, suggested in a government report from 2012 [30], and thereafter used as a basis for cost-benefit analyses in Norway.

To arrive at our estimate, we used the method suggested by the Directorate of Health [29]: first adjusting the VSL for inflation to 2021 (33.35 million 2021 NOK; €3.34 million [27]) and then adjusting the VSL to a healthcare perspective (28.90 million 2021 NOK; €2.89 million [27]) by removing the production costs (the cost of lost production in case of mortality or morbidity). In the estimate by [30], the expected remaining life of an individual which is saved by the risk reduction is 40 years. However, Lossius et al., have estimated that the expected number of life years gained when the increased survival chances are attributed to HEMS, is approximately 7 years [33]. The value €2.89 million was therefore adjusted to €813,911 to account for the shorter life expectancy of persons saved by HEMS.

## Results

**Fig 2:**
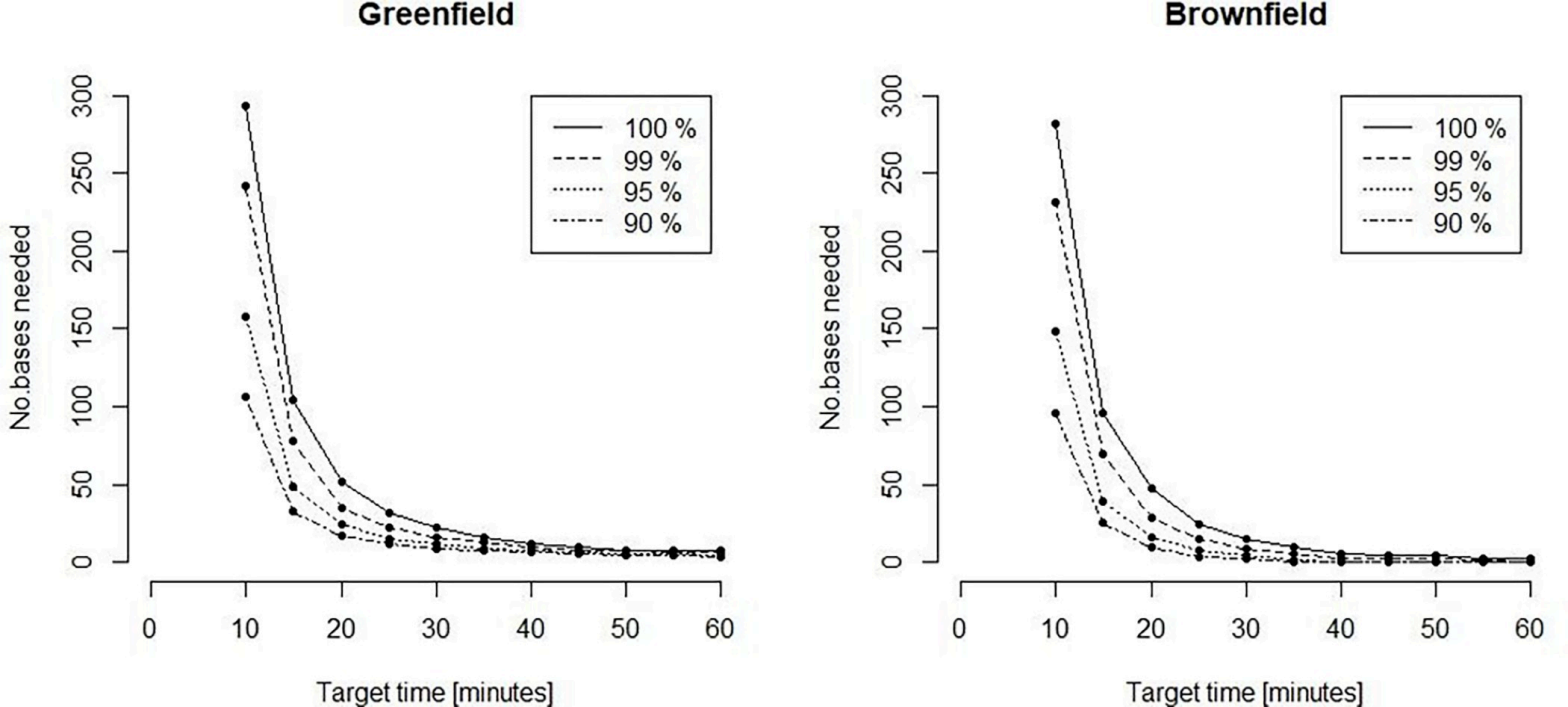
Number of bases needed to cover various percentages of the population.

**Fig 3:**
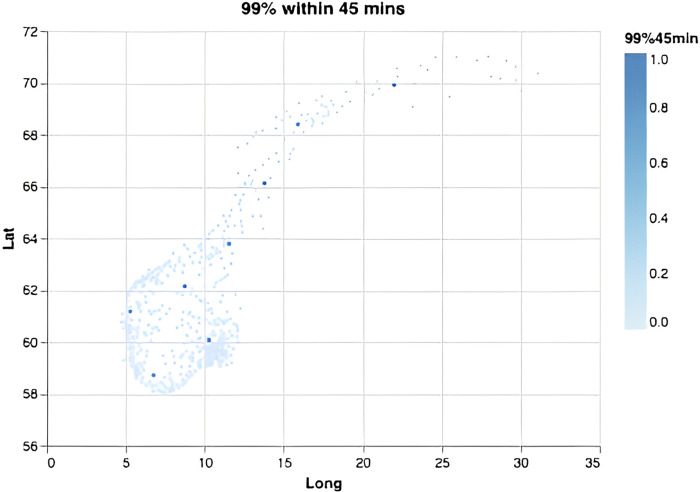
Optimal base locations. For a scenario where the goal is to cover 99% of the inhabitants within 45 minutes, this requires 8 bases.

**Fig 4:**
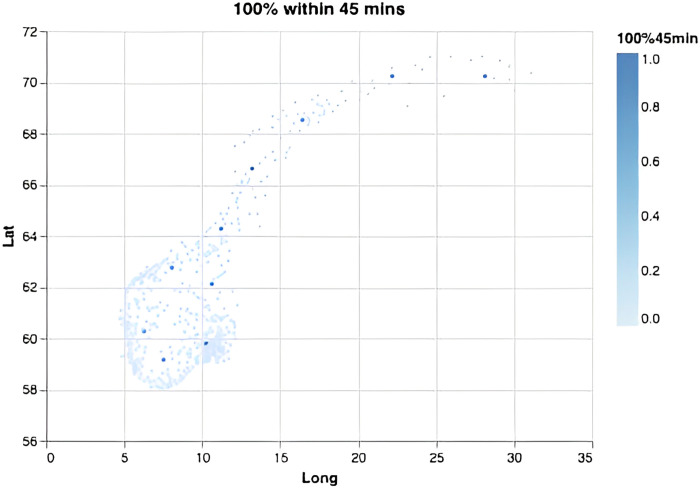
Optimal base locations. For a scenario where the goal is to cover all inhabitants within 45 minutes, this requires 10 bases.

**Fig 5:**
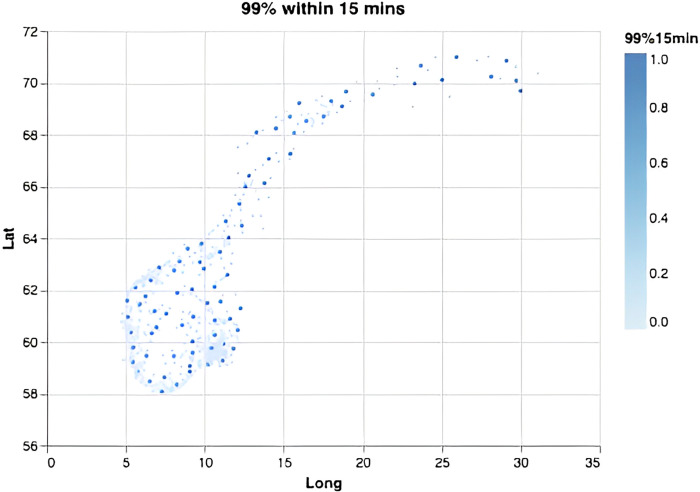
Optimal base locations. For a scenario where the goal is to cover 99% of the inhabitants within 15 minutes, this requires 78 bases.

**Fig 6:**
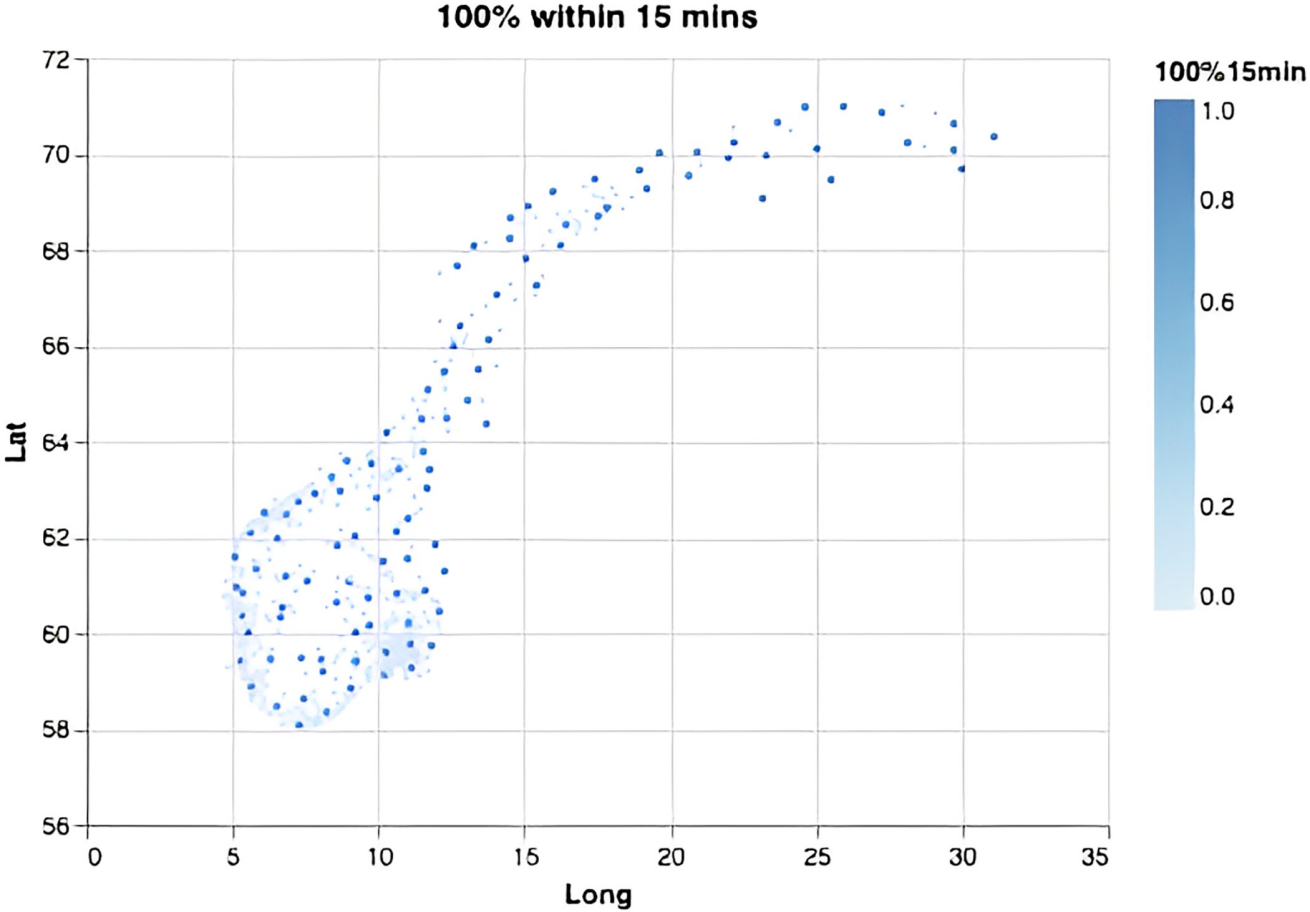
Optimal base locations. For a scenario where the goal is to all inhabitants within 15 minutes, this requires 104 bases.

We computed the required number of bases depending on the response-time target and the fraction of the population required to be covered (Fig 2). Overall, each new base adds increasingly less to the overall coverage, and steadily more bases must be added to achieve the same effect in increased coverage. This effect is especially visible for small time thresholds and large coverage percentages, demonstrating the non-linear nature of the problem. Notably, in order to serve 99% or 100% of the population within 45 minutes one only needs 8 or 10 optimally located bases (Figs 3 and 4). This is fewer than the current 13 bases. The locations of those 8 or 10 bases would, however, need to be completely re-organized as compared to the existing base structure (Fig 1). When lowering the threshold to 15 minutes a dramatic increase in the number of bases is needed (Figs 2, 5 and 6). Covering 100% of the population within 15 minutes requires 104 bases, spread in a fine grid throughout the country (Fig 6), with each base being on average just 34 km from its nearest neighboring base.

These results are further detailed in Table 1, summarizing a range of different scenarios, with response-time thresholds ranging from 15 to 45 minutes, covering 99% or 100% of the population.

**Table 1:** Number of bases and personnel needed to cover different percentages of the population for different time thresholds.

Coverage	Time threshold (mins)	Number of bases	Running costs (million €)	Personnel needed	Lives needed to save	Expected number of missions per base (min-max)	Population served from the least efficient base	Incremental costs (million €)	Incremental lives needed to save	Incremental need for personnel
100%	15	104	551	1456	678	3–242	939	276	339	728
	20	52	276	728	339	4–327	1403	106	130	280
	25	32	170	448	209	9–783	2726	53	65	140
	30	22	117	308	143	9–817	2726	32	39	84
	35	16	85	224	104	38–935	12282	21	26	56
	40	12	64	168	78	99–817	31631	11	13	28
	45	10	53	140	65	107–703	34357	-	-	-
99%	15	78	413	1092	508	12–815	3868	228	280	602
	20	35	186	490	228	57–921	18266	69	85	182
	25	22	117	308	143	84–825	26979	32	39	84
	30	16	85	224	104	84–572	26979	16	20	42
	35	13	69	182	85	99–622	31631	16	20	42
	40	10	53	140	65	242–297	77340	11	13	28
	45	8	42	112	52	543–900	144910	-	-	-

Values are estimates *per year* in a greenfield situation.

* Incremental estimates from the lowest response-times (45 minutes) are not included. Incremental numbers are estimated in comparison to the row below (i.e., a response-time difference of 5 minutes).

### Estimated medical demand

We express the estimated medical demand as the expected number of yearly missions per base. As some bases will be more busy than others, this provides a range of numbers; the minimum and maximum are listed in Table 1. For example, when covering 100% of the population in 15 minutes, the least efficient base performs just 3 missions per year, while the busiest base performs 242 missions per year. These numbers are computed under the assumption that each incident is served by its closest base and a yearly total of 16,246 missions nation-wide.

### Healthcare personnel, healthcare costs and benefits

The necessary number of bases directly translates into estimates of need for personnel and healthcare costs, as indicated in Table 1. For example, with 6 doctors needed to man a HEMS base 24/7/365, covering 100% of the population within 45 minutes, would require 60 anaesthesiologists. However, decreasing the response time to 15 minutes for 100% of the population would require 624 anaesthesiologists. If including pilots and paramedics, the total number of personnel required for the above-mentioned scenarios would be 140 and 1456, respectively (Table 1).

Likewise, the healthcare costs of reducing the response times increases nonlinearly with increased coverage and reduced response times. For example, a response time of 45 minutes for 99% of the population would require 8 bases with an expected healthcare cost of €42 million. Reducing the response time to 30 or 15 minutes would increase the required number of bases to 16 and 78, and correspondingly increasing the expected healthcare costs to €85 million and €413 million, respectively.

The number of lives needed to save increases drastically as the time threshold decreases (Table 1). For example, in the 100% coverage scenario, reducing the time threshold from 45 to 40 minutes requires just 28 extra lives saved, while reducing the time threshold from 20 to 15 minutes requires 728 extra lives saved yearly. There is also considerable difference in the incremental number of lives needed to save between the 99% and 100% coverage scenarios. For example, to achieve a NSB of zero, reducing response times from 20 to 15 minutes requires saving 602 additional lives (99% coverage) or 728 additional lives (100% coverage).

## Discussion

The primary aim of the study was to estimate the number of HEMS bases needed in order to reach different proportions of the Norwegian population (99–100%) within varying response-time targets (10–60 minutes). Further, we estimated the corresponding need for personnel and accompanying healthcare costs, translating those to the number of lives that would need to be saved for the total system to be cost effective. Our findings indicate a nonlinear relationship between reduced response times and the number of HEMS needed, and, correspondingly, so is the need for personnel, healthcare costs and number of lives needed to save.

Using a mathematical model for base locations allows for experimenting with the number of bases and their locations, exploring optimal solutions for different scenarios of interest. The MCLP model applied in this work is well established within the logistics optimization literature [30, 31], but has so far not found widespread use in health economics. Given the possibility of quantitative experimentation such mathematical models allow for, such models should find more uses within the field.

Any mathematical model makes specific assumptions about the problem at hand. The MCLP implicitly assumes that whenever a patient is in need of HEMS, a helicopter is available at the closest base. The model is thus sometimes described as a best case scenario [15]. When there are few bases this assumption is likely to not hold, as there might be concurrent demand. Then a somewhat more sophisticated mathematical model like the Maximum *Expected* Covering Location Model (MEXCLP) [32] might be a better modelling choice. For situations with many bases, however, this probability of concurrencies is very small, with bases and helicopters being abound. This is the case when the response-time target is low and the coverage is high–as is the primary target for our analyses–and then the MCLP is a sufficiently advanced model.

In our analyses we assumed that the only changes that would occur to the healthcare system are the number of HEMS bases. However, in reality, such an expansion might have led to a range of other changes within the healthcare sector, for example, better access to HEMS might reduce the need for acute care in some small, rural hospitals–thus, influencing the health not only of the persons treated by the HEMS, but also patients with less severe conditions treated at these small, rural hospitals. Similarly, when centralizing health care services, the accompanying reduced acute care in rural areas can be compensated by expanding HEMS. None of these spill-over effects are modelled, and the simplified picture of this expansion should be kept in mind when interpreting the results. Future models can be built to answer more complex questions, such as queuing and competing interventions, building on the current model.

Because of limited knowledge on the expected effect from the HEMS expansions modelled in the current paper, our aim was not a precise estimate of the cost effectiveness but to indicate the necessary effect (lives needed to save) for the HEMS-expansion to be cost effective. However, we can attempt to assess when the expansion is cost effective: a base, when considered in isolation, requires saving approximately 6.5 lives per year to be cost effective (€5.2 million /€0.8 million (VSL)). This number cannot readily be compared to the number of missions per base (in Table 1), as the question remains how many of the missions are life-saving ones. Lossius et al. estimated that out of 447 missions carried out by helicopter, 45 patients (approximately 10%) received life-saving treatment [33]. This means that a base would have to have 66.5 missions per year to save 6.5 lives. Given the current demand in Norway, yearly 0.3% of the population (16,246/5,415,166) requires HEMS treatment. Using this, we can estimate that a base would need to serve a population of at least 22,116 inhabitants (66.5/0.003) to save sufficiently many lives for the base to be cost effective. From Table 1, we see that even the quietest base still has sufficient missions in the scenarios covering 100% in 40 minutes, or 99% in 25 minutes. These simple estimates rest on a range of assumptions regarding availability of helicopters, severity of disease in the population covered, no competing interests etc. Also, it rests on the assumption that *all* HEMS-bases should be cost effective; the ethical philosophy of utilitarianism. We could also argue for a system where decision makers could accept a lower efficiency in some bases because of equity considerations (i.e., egalitarianism), as long as the *system as a whole* is still cost effective. To that end, focus on the most expensive scenario in Table 1: 104 bases serve 100% of the country within 15 minutes. That would mean the system as a whole is able to serve the total demand of 16,246 missions per year, containing an estimated 1636 life-saving missions, which comes down to an average of 15.7 lives (1636 /104) per base, per year. This estimate is above the required 6.5 lives per year to break even. However, in reality many of the 16,246 missions would occur in the large cities and could thus be served by ground-based ambulances. More research is needed on the expected effect of helicopter expansions before a conclusion regarding cost effectiveness can be reached.

From a patient perspective, immediate access to highly competent health professionals as well as rapid transport is purely beneficial. Although the costs associated with a total national coverage in 10–15 minutes are high, the total number of lives needed to save appear to be of corresponding magnitude. Despite this analysis mainly focusing on costs, the feasibility of such a substantial service expansion needs to be addressed. In order to provide sufficient personnel running the service according to mandatory legislative requirements, a substantial number of personnel would have to be employed. This in turn would likely impart several effects on costs (e.g. training, licensing, certifications), availability (e.g. limited number of personnel available) and fulfilments of legislative requirements (e.g. pilots need a certain minimum of flight hours per year). In the current service, about 14% of available anaesthesiologists in Norway (150 of 1,086 anaesthesiolgists) are already working as HEMS doctors, which is already affecting strained resources [34, 35]. Also the high requirements of Norwegian HEMS pilots are affecting the number of available resources. We have also shown that in a maximal covering situation (100% coverage within 15 minutes), some bases would have a number of missions as low as 3–4 per year. Such low numbers would not provide adequate exposure to critical procedures and would therefore necessitate further in-hospital training to obtain a desired professional competence. For all of these reasons, a drastic increase in HEMS would likely prove difficult to implement. Moreover, secondary effects on other services (e.g. competing for personnel) would need to be taken into consideration.

### Strengths and limitations

We did not take into account that shorter response times may lead to improved health effects in patients who would also have survived with longer response times. These effects were not incorporated for simplicity reasons. In this way, our estimates of the benefits are rather conservative.

Our mathematical model analysed the HEMS as a stand-alone resource of the healthcare system. For completeness, however, one would have to take into account the involvement of connected resources such as ground ambulances and other health facilities, in addition to factors that directly affect the accessability of HEMS (hazardous weather conditions that result in increased response times, increased flying distances and a larger risk of mission rejection). This might affect the number of HEMS bases and helicopters needed to cover the full population.

The use of population data as a proxy for demand is also debatable, as previous studies have shown that incidence and population densities are poorly correlated [3]. Using historical HEMS demand might improve the estimates for our 99% coverage scenarios: this could potentially better grasp which areas are more likely to contain severely injured people (one can imagine a higher likelyhood in for example skiing areas). However, this approach has its own disadvantages because the historical data is biased: it only contains the requests that were actually accepted. The results for 100% coverage would not be affected by this choice.

In this study, we only estimated the potential effect of lives saved. However, we acknowledge that to evaluate whether (health) investments in Norway are worthwhile, the suggested measure is quality-adjusted life years (QALYs) gained [36, 37]; that is, that both the length and quality of life should be incorporated. At this point, neither the expected number of lives saved nor QALYs gained are used explicitly when Norwegians decide whether and where to build HEMS bases. Therefore, our approach of connecting these investments to lives saved is already a step in the right direction.

We studied whether the Norwegian HEMS system *as a whole* would be cost effective (have a NSB of zero). However, if one is interested in the effectiveness of the least-used base, insights could be derived from the column *Expected number of missions per base* in Table 1 and focusing on the lower end of the range provided there.

We showed that reducing HEMS response times to 10–15 minutes requires a drastic increase in the number of HEMS bases across Norway. However, it may be possible to achieve a number of lives saved such that this investment is justified.

Building HEMS bases is far from the only way to improve saving lives in rural areas. Alternate ways include schooling, CPR training and the placement of defibrillators and deciding to build a new base should include considering these.

## Conclusions

Reducing Norwegian HEMS response-time targets from the current 45 minutes covering 90% of the population to 10–15 minutes covering 100% of the population requires a drastic increase in the number of HEMS bases. While the current target is met with the current 13 bases, reaching 100% of the Norwegian population within 15 minutes requires 104 bases. For 99% coverage 78 bases suffice. This increase in number of HEMS bases comes with an increased demand for personnel. Reducing the response-time threshold from 20 to 15 minutes for 99/100% of the population yields an incremental need for personnel of 602/728, with an accompanying incremental cost of 228/276 million EURO per year. Whether this can be considered cost effective depends on the choice of ethical philosophy; utilitarianism or egalitarianism. An egalitarian point of view–that is, requiring the HEMS-system *as a whole* to be cost effective although the least efficient bases need not be–implies a yearly total of 280/339 additional lives would have to be saved to obtain a net social benefit of zero.

## Supporting information

S1 Data Coordinates. (XLS)

S2 Data Flight times. (CSV)

S3 Data Existing bases. (CSV)
